# Conservative Treatment of the Dental Pulp

**Published:** 1893-10

**Authors:** 


ARTICLE II.

### CONSERVATIVE TREATMENT OF THE DENTAL PULP.

Dr. Robinson.—I received a note from Dr. Dickinson saying that he would like to have me open the discussion on his paper. I am not prepared to give very much of a talk upon it, except as to my own views on the subject. I will say, at the start, that I have never been very much of a believer in the conservative method of treating the pulp, that is as regards trying to save the pulp after it has been fully exposed. I may agree with the doctor in certain cases where it is advisable. I believe in trying to protect the pulp from thermal changes, and I presume every dentist present also believes in it. The doctor states some cases, which he thinks are favorable cases for conservative treatment of the pulp, and among others those in which the pain has been caused by a pressure of food or various foreign substances against the pulp. I take issue with him on that point. If the pressure has been sufficient to cause decided pain, I believe it has been sufficient to press the pulp out of its proper relation with the tubuli of the dentine. We believe, although it has not been fully proven, that there is a vital connection between the pulp and the dentine. We have that demonstrated to us every day in the sensitiveness of the dentine. If this pressure has been sufficient to disturb the relation of the parts, that, will they be re-established is a question in my mind. I think they will not. If the pressure has been slight, and the pain has not been severe, I believe it can be done as the doctor stated. Speaking about the sensitiveness of the bubulous portion, if there is a central hyperæmia of the pulp at the point of exposure, we know that there is extreme sensitiveness to cold applications, cold water, etc. In such a case, if this hyperæmia can be reduced, I think there is no question but what the pulp can be saved if handled properly. If the pulp has been allowed to remain in this condition, and this hyperæmia has gone on until it verges on congestion, that tooth will be sensitive to hot applications. You will notice a difference in the stages. At one time it is more sensitive to cold, and at another time to heat. I think where they are more sensitive to hot applications that there is less chance of saving pulps than when they are sensitive to cold ones. It seems to show that the hyperæmia has gone on until it verges on congestion. There is more fluid in the pulp and in the surrounding tissue, and therefore there is less chance, to my mind, of saving the pulp. We often hear it advocated that even parts of the pulp can be saved, even after the bulbous portion has been destroyed. I do not believe in that theory. I believe if the bulbous portion of the pulp has already been destroyed, the best we can do is to remove the balance, and to fill as perfectly as possible. I do not think that after the relationship between the dentine and the pulp has been disturbed, and so cut off, that there is any use in trying to save what is left of the pulp. Of course, the doctor did not advocate that in his paper, but I am simply speaking of it in a general way. I think the method is as good as any advocated at the present day. I have nothing different in the way of method, where I do attempt to save the pulp. It is very seldom though that I try to save the pulp, unless the exposure is an accidental one, or unless everything is favorable.

I think, perhaps, that I go to the other extreme sometimes, and destroy pulps that might have been saved, perhaps on the principle that "dead men tell no tales," and perhaps because I doubt my own ability to save them. I have seen so many cases where pulps have died under temporary fillings and under permanent fillings, and still gave no trouble, that I am led to believe that in many of these cases that are spoken of as successful, the pulp has really died. On the other hand, we find, in our attempts to destroy the pulp sometimes, that there seems to be an endless amount of vitality in it. It seems as if anything in the way of protection would conserve such a pulp, inasmuch as we find almost endless difficulty in trying to destroy it. We find when we tell the patient that the pulp is destroyed, and he can use it without hurting, that he will doubt our word. There is nothing further for me to say, Mr. President. I have not given the matter much study and so cannot open the discussion as I would wish to.

Dr. Reid.—I would like to ask Dr. Dickinson a question. He states that the preparation which he uses is about the consistency of putty. If that is so, how do you make the application ?

Dr. Dickinson.—I experienced a little difficulty as to how to express that. Now in almost all discussions it is spoken of mixing the phosphate cements to about the consistency of cream. The idea I had was not that it should be of the consistency of putty used by painters. Of course it would not be expected to be made too hard to spread. I did not know of any other term that would express it. It wants to be in a rather soft state in making an application for protection.

Dr. Reid.—I supposed when the doctor made the remark, that it was a term he did not want us to take literally, because I do not think that he or any one else would employ any substance of that consistency. While I agree with the paper in the main, there is one thing I wish to add. If there were more care, more nicety and attention given, there would be a great many more pulps conserved than there are. The trouble originates in more than one way. Many times you get a patient in that condition and they become exceedingly impatient, they want to get rid of the pain or trouble, and too frequently we give way to them for that very reason. We want to satisfy them and get them free of that trouble. Now, in the application of anything to cap a pulp, I feel you must have some substance that will adapt itself as easily as possible to the cavity. I cannot put my hand on it, and I want to get something that will adapt itself to that cavity.

Dr. Dickinson.—When I was speaking of pulp capping I said it must be much thinner so as not to require any handling.

Dr. Reid.—I have used the doctor's preparation some. I will confess, as he said, that the trouble comes from lack of proper handling. I have been troubled to get it to work right. I suppose he has worked at it, and in mixing it up can get a consistency that I have not been able to get. I think that one who uses almost any material will become more or less dexterous in the use of it, and also in the use of the second finger. I do not know but what the preparation is just as good as any, but I feel this way: I think, as Dr. Robinson says, there are too many of us a little anxious to bury the pulp as soon as we can. I feel that all of us should pay more attention to a thing of that kind and try to save all we can. Even if we only get one or two out of a great many, I think we have gained that much at least. After all, there is a great deal to be gained by the delicacy and nicety with which you do these things. As the doctor says, you must use a rubber dam, and if the pulp is in a position where you cannot see it, you had better open the tooth up and work till you can get at it. It is an operation that requires you to get where you can see everything, and do in the best manner.

Dr. Robinson.—I would like to ask the doctor one question. I understood him to say that after flowing this thin preparation over the exposed pulp, then he follows with the oxyphosphate or oxychloride. Do you place a metal cap between ?

Dr. Dickinson.—I do not. I sometimes use asbestos. Prof. Weeks uses asbestos almost entirely. He uses it for convenience sake.

Dr. Robinson.—The reason for asking was simply this. Any of us who have had occasion to remove a crown or bridge, that has been in the mouth for several months or a year, as the case may be, have found in removing the oxyphosphate from the cap, a condition of things that is not desirable. The oxyphosphate seems to have absorbed everything that is applied in the mouth. It seems to me that demonstrates that oxyphosphate, or any of our cement filling, will take up more or less of these gases, and more or less of this poisonous product or decomposition. Now if they will do that under a crown, even if the crown is pretty closely adapted to the tooth, will it not occur within a tooth, and would not the putting of a metallic substance or something similar between this cap and the oxyphosphate following, have a tendency to keep everything from the pulp itself? That is the idea I wish to bring out.

Dr. Dickinson.—I hope, Mr. President, there will be a full and free discussion of the subject. As I said before, I am somewhat of an enthusiast about this matter, and the conclusions I have come to are based upon rather longer experience than things of this kind usually are, that are brought forward at these meetings. I am not at all sensitive to criticism, for I want to get at the truth, and if I am making any mistake, I want it brought out. I will be glad to give any help that I can. That is what I am here for. Now, in regard to using the oxyphosphate over a capping. It is, I may say, the principle use of it. It is simply to get a covering of something that will harden immediately so we can go along with our work. It is not necessary in a cavity of ordinary size to put in that covering there. The dehydrating of the tooth itself, with alcohol, is antiseptic, and the chloroform is antiseptic. The essential oils put into it are antiseptic, and the heat which is used to drive these out, they all act in an antiseptic way. Now, the mixture of the oxyphosphate, and especially with the iodoform in it, will take up what little of the noxious gases that remain in the cavity, that the Doctor spoke of. The oxyphosphate is simply used to make a hardened vitreous layer—of course it is not exactly vitreous, because it is not hard enough for that, but it makes a covering so we can go right along with our work.

Dr. Van Duzee.—I would like to ask the doctor a question before the society, because I think it is one we may all come across. I have used the preparation a couple of years, and have frequently found that the patients complain of still tasting that medicine that I put in under the filling, even after the lapse of a month or two. Now I first thought in one or two cases, that perhaps I might have been careless enough to insert my fillings in such a way that there might be some leakage, but the fillings stand nicely, and I cannot find any evidence of that sort of trouble, so I am led to believe that some of the ingredients that are used in the preparation are absorbed by the pulp, and are eliminated through the mucous memrane of the mouth, which produces that taste. It seems to be almost constantly in the mouth for months after the material is used. Now, it seems to me, it would not be possible to have leakage enough around a gold filling to allow that to escape without some fault being brought to the surface in the filling itself. I would like to know if the doctor has ever come across that condition of affairs.

Dr. Dickinson.—I have occasionally had complaints of that kind, but very seldom considering the amount that I use of the oxide of zinc and the iodoform with the menthol. There is so little of it that I cannot conceive of its getting around in that way at all. When we treated the condition that we speak of as ulcerated tooth, with a combination of creosote and iodine, we used to constantly hear of that. Even if we got the tooth in a good condition and filled it, the patients would complain for months and months that they could taste it. The proportion of the iodoform with the oxide of zinc is so small, that I hardly think the iodine would be enough to produce that, and I do not think the menthol would do it. I cannot account for it.

Dr. Van Duzee.—Have you ever met with that condition in your practice ?

Dr. Dickinson.—Yes, occasionally, but not very often. It has been so seldom that it has not been a factor in taking into account the objections against using it.

Dr. Stearns.—There is one point on which I wish to disagree with the doctor, and that is, in the use of medicines to aid the operation of capping. I believe it is theoretically and practically wrong. My opinion is that all treatment should cease at the time that the pulp is covered. That is the end of the treatment. If we have not got it in a condition to be covered, we had better not cover it. In my estimation, the ideal pulp covering or cap would be a finely glazed piece of porcelain. Of course we cannot use that and the next thing is to get something as nearly approaching it as possible. I think in all cases of this kind, that the application of any medicine, sealed into the cavity, is deadly wrong. Furthermore, I think it is not necessary—I am somewhat of a crank upon this subject myself. I will say that within the last year I have used arsenic twice that I know of and possibly three times, but not more than that, and yet I have had more cases than that of exposed pulp. I almost invariably make the attempt to conserve the pulp and in a large proportion of the cases I am successful, because I make it a rule that whenever I cap a pulp I let medication alone.

President Merrill.—I would like to ask Dr. Van Duzee if in the cases he spoke of, of tasting the application after it had been inserted, if that occurs immediately after the filling has been inserted, or some time afterward, and if it was not possible that the pulp may have died under that application and the medicine passed out through the apical foramen, and be tasted in that way.

Dr. Van Duzee.—I do not think there is any question but what the pulp is alive and in good condition, for this reason: I have yet to find more than one or two cases where the treatment has been anything but very successful. Now, in those cases, although that is a point that I never thought of before, as I look back upon them it seems to me that there is nothing in that supposition. I think the pulps are alive and healthy because I do not think we could have death and disintegration of the pulp, and the passage of a foreign substance through the canal so easily, without there being some other indications of trouble. So far as Dr. Stearns' point in regard to leaving medicines in the tooth is concerned, I would like to ask him if there is any good reason why you should not insert under a filling a suitable filling substance saturated with a drug which is known to be rather favorable in its action toward the pulp than otherwise, and allow the pulp to absorb all that, leaving the menthol and the materials of the preparation of zinc, to occupy the space. It is a porous substance to be sure, but I do not understand why it is not perfectly proper to leave underneath a gold filling a mixture of that kind. I am favorably impressed with the results I have obtained from the use of that drug. I have used it in the very worst cases I could possibly have undertaken to handle, and have obtained results which were very satisfactory.

Dr. Jennison.—I must confess that my enthusiasm in regard to the conservative treatment of the pulp is not very great. I suppose we are all governed somewhat by experience, and we may be largely at fault ourselves sometimes for the obtaining of good or bad results. Where any pulp has been in a pathological condition, I doubt very much its being brought back to a perfectly normal state where it will remain so. Where they do occur in that way, the first treatment is always to saturate them with some antiseptic, then go ahead and cap them, and they remain quiet. The question is what is the state that they are in. Now there is often enough in the antiseptic itself, to prevent decomposition from setting in very much, and a great many times these operations get the credit of succeeding beautifully. Still I think that sometimes you will find that the pulp was dead or nearly so, if the true state of affairs could be learned. The antiseptics with which they are saturated preserve them from active suppuration. Of course a recent exposure, if it is carefully capped, acts very much better because you have different conditions to contend with. But why do we want to save it? If, as Doctor Sudduth, and a number of other authorities claim, it is simply a formative organ, what do we need it for after the tooth is formed.

I do not think we find a very decided change in the structure of the tooth if the pulp is carefully removed and the canal filled with some antiseptic material. It seems to me that the principal point we are aiming at is to avoid an infectious trouble from a dead pulp. Antiseptics, I hope, will some day relieve us of that danger, and bring us to where we will have no abscesses and no inflammation if the pulp is thoroughly removed. But that is not done in many cases, and for that reason, with the limited knowledge at the present time on the question of antiseptic treatment, many of us are forced to the conservative treatment of the pulp. I attempt it in many cases where I feel I cannot thoroughly cleanse the canal, and I think many others try it for the same reason. When we can thoroughly remove the dead pulp and find some more satisfactory way of devitalizing, I think we will hesitate a great deal less about these different points. Until we do, we have to a certain degree, to adopt the conservative treatment and in that case the method before the convention is undoubtedly the best. I must acknowledge that I have capped a good many of them with arsenic, and use antiseptics thoroughly afterward, and although I cannot prove it by figures, I think the results average about as well as where I attempted the conservative treatment in questionable cases.

Dr. Leonard.—My experience has been similar to that of Dr. Jennison. I have had no particular success with capping exposed pulps with the idea of keeping them alive in the tooth. I think the ideal antiseptic treatment of a pulp that has been devitalized, is such a treatment as will keep the tooth from inflammation and ulceration when some portion of it is still in the tooth. We all know that it is impossible to get all that pulp out of the tooth in very many cases, and those are the ones that give us the trouble. If we can get a good straight canal where we can get the pulp out readily, there is no trouble, but where it is impossible to do this, then the thing to do is to find some antiseptic that will keep that condition of affairs from causing trouble. I have had very much better success with devitalizing the pulp than I have had with trying to preserve it alive after it has been really exposed.

Dr. Goodrich.—I rise to return thanks for the paper, and also to call attention to the admirable capping material of Dr. Dickinson's, which is on the market. It is about the only thing I use, and I have met with very good success in using it. In regard to the taste that Dr. Van Duzee spoke of, I think he will find that it is a sort of peculiarity among peculiar people, and I think it ought to be looked at in that way. I do not think there is very much in it mysely.

Dr. Brimmer.—I do not see that simply putting a medical preparation under a filling does any harm, as that pulp itself is a circulating medium. I cannot see why the pulp cannot perform the function peculiar to itself like any other part of the circulation. I do not see that putting a medicine under the filling is going to endanger that pulp simply from the fact that the medicine is there. Oftentimes a person suffering from an ulcerated tooth on the lower jaw does not go to a dentist, and the pus discharges itself. Now what becomes of that pus? Why, it is carried off by absorption in the circulation. We do not always realize what an enormous amount of work is done by the principle of absorbtion, and I do not see why the pulp cannot do a little of that work. Dr. Jennison made the statement that the function of the pulp was simply formative. If that is the case, I do not see why we should find in the case of an old gentleman, for instance, who had a tooth out, and yet there was a live pulp there. It seems to me that if the function of the pulp was formative, that when the tooth became calcified the function would cease. I do not think that we ought to obliterate something that was put there by a pretty good workman.

Dr. Jennison.—In a great many cases if we examine the teeth in elderly people we will find that the pulp cavity has very decidedly decreased, and in old age, has disappeared entirely in a very large number of cases.

Dr. Brimmer.—In excessive attrition?

Dr. Jennison.—Yes if that is contrary to the design of the Creator why was this extra dentine created to fill up the pulp?

Dr. Brimmer.—It is mechanical. In excessive attrition, when the position of the pulp recedes, of course it calcifies. When you find there is no attrition, no wearing down of the parts, I think you will find a pretty healthy pulp, and I should want to go rather slow. When we find attrition, of course we look for recession of the pulp.

Dr. Jennison.—I have one case where I think every tooth in the patient's head is affected with the receding condition of the pulp, and yet there is scarcely any wearing away by attrition. The pulp is about half receded, that is, in every tooth I have opened.

Dr. Reid.—I want to ask Dr. Dickinson another question. He may think it a little unfair. What would be your treatment, in the case of an accidentally exposed pulp?

Dr. Dickinson.—It is presumed in the first place that you have that cavity isolated from the fluids of the mouth. There will be a little hæmorrhage. I would have no hesitation in going right on and capping this just as I stated in the paper.

Dr. Reid.—There is one of the cases where I will agree with Dr. Stearns. It looks as though there was no earthly sense in medication. The supposition is that it is perfectly healthy, and is merely an accidental exposure. It that case why do you medicate it? A doctor whom you all know well, talked that doctrine to me, to turn around and use creosote or carbolic acid on that fresh exposure. I have always wanted to ask him if he cut his finger, would he treat it in that way. Now if you can get something that is non-irritative and close it up without medication it is all right. You would not want medication if you simply wounded your finger, but would close it up and it would heal by first intention.

Dr. Brimmer.—I think it is claimed that it is just what they want to use—carbolic acid. It produces an artificial membrane and keeps the air away from the wound. Nothing will ever heal until nature throws out a membrane to shield it.

Dr. Dickinson.—In the first place, Dr. Reid has spoken of the preparation as a medicine. It is simply a semi-cement. It is not a cement in the sense in which we ordinarily use the term. It is absolutely non-irritative; it is bland; it is something that the pulp of the tooth will tolerate in contact with it. When you expose the pulp accidentally in an excavation, it is very seldom a large exposure. If you could compare it with anything we could speak of, it would not be as large as the end of a cambric needle, ninety-nine times out of a hundred, and the natural retraction with perhaps a little application on there, will enable nature to take care of that without anything else.

Dr. Van Duzee.—There is one thought that came to me which I would like to mention, and that is, if you open a pulp with an excavator, you have not got a clean exposure, but one that is contaminated by an instrument that is loaded with the very germs that destroy the tissue.

Dr. Reid.—I would ask Dr. Van Duzee this question. Where you get this tough leathery decay, where you have exposed this pulp and the instrument has never come in contact with the nerve, but still lifted off that decay, what would be the effect?

Dr. Van Duzee.—It is the same in either case. The leathery decay is filled with the same matter.

Dr. Spaulding.—I fully agree with what has been said by some of the doctors in regard to conservative treatment, though I have never had very much success with it myself. I believe that in some cases it may be done, that is to save a fully exposed pulp. The pulp, as we understand it, is a fleshy substance confined within the walls of the tooth, and when it becomes exposed and inflamed, if at all inflamed, there must be swelling. During the inflammatory stages of the pulp, if it is capped by anything, after being medicated, the inflammation sudsides and the pulp is supposed to draw in its horns, so to speak, and assume its natural position. There is a vacuum there, slight it is true, but nature abhors a vacuum, and there is something wanting there. The pulp, if inflamed, should be treated before it is capped; before it is covered entirely it should be treated until it is in perfect health. Of course there is a very fine line to be drawn there. When the pulp is perfectly healthy it may be covered, but if the chances are against its being healthy, on the principle that "dead men tell no tales," as Dr. Robinson stated, I like to remove it entirely. Yet as Dr. Leonard said, the proper treatment for the pulp that has been removed either entirely or a portion of it, we know that in certain surgical operations the nerve still remains in many of the wounds although it has been severed, and if that remaining pulp may be restored to its normal condition, it may be covered, if covered without pressure. Dr. Stearns, in speaking of a proper capping for the pulp, mentioned porcelain, and Dr. Robinson spoke of some metallic capping. I hardly agree with those gentlemen in the capping of the pulp with a metallic cap, as it is a very delicate thing, and it is necessary to get the exact contour of that cap in order to protect it, and not have a vacuum underneath. If there is a vacuum, what are you going to fill that vacuum with? If the porcelain capping exactly fits, I may agree with the gentlemen, that it is as good as anything we can put on, but the better way would be to cover that with something as Dr. Reid has said, that would flow over it without pressure, and resume the covering of that pulp without pressing. Then it may be covered and sealed up with cement. I should hardly think of putting in a gold filling into a tooth where there has been such treatment, until I was perfectly satisfied there was not going to be any trouble following. I should fill with cement. Dr. Robinson was speaking of the cement causing trouble, or absorbing that which it should not. That I think depends largely upon the kind of fluid that the oxide of zinc is mixed with. Some of the mixtures disintegrate and give off a very bad odor. I have removed crowns where the cement was as solid as stone, and had no odor whatever. I have no doubt that we have all removed cement fillings in teeth where the cement has hardened and become perfectly solid, and was entirely free from any odor, when removing a portion for the purpose of covering the capping that has already been put in. I feel sure myself when there is a full exposure, and when there has been pain in the tooth and decay clear to the nerve pulp, that the pulp should be removed. In the case that Dr. Reid spoke of where the pulp has been wounded, I should simply touch it with camphor, the alcohol in the camphor will evaporate leaving the camphor itself, which is very healing and cleansing. One may follow the camphor treatment with a little oxyphosphate, or cover it with gutta-percha, and go on with his filling.

Dr. Dickinson.—The discussion has taken a somewhat wider range than I expected, but I am glad that these views of the different members have been brought out. The premises, however, upon which some of these statements have been made, were not justified by anything that was said in the paper. The first statement I made was that the cases should be selected. I didn't say that it was justifiable or advisable to cap any pulp, if it gave pain, excepting such pain as could be immediately relieved. One gentleman made the remark, that where the pressure of food was on it, the condition would be unfavorable many times for this operation. Now, when I was speaking about the pressure of the food in the cavity causing pain, I was not speaking particularly of exposed pulps, for we know there are many pulps not fully exposed where pressure in the cavity will cause pain for the time, but it will be instantly, or very soon, relieved if that pressure is removed. I said distinctly that no treatment would take the place of the most discriminating judgment in these cases. I also said that there were cases in which I would not attempt conservative treatment at all, that devitalization was the proper method. I do not think, in a dicussion of a subject like this, that it is exactly scientific to say that the ideal pulp capping would be a certain material that perhaps the gentleman had never tried, or never experimented with. I might have gone on for fifteen minutes in enumerating the different articles of which I know, and which I can refer to in my library, as having been tried. I

simply took a number of those as indicating that the field had been worked. I think I could name thirty or forty agents that had been used in filling pulp canals. While the discussion has taken a wider range than I looked for, it has not been in every respect confined to the paper. It has brought out many things which I hope will be beneficial to us all, if it does not do any more than to set us all thinking. Now my own personal success is what has made me enthusiastic, and what I have done, others can do. It does require the most delicate manipulation, I will say, especially in capping exposed pulps. Even those which are most favorable will not admit of bungling work. Of course, as I said, I am aware of the diversity of opinion in regard to where there is any use in trying to save a pulp at all, but I took the other side—that it was. If any one is satisfied to devitalize every pulp that is nearly or fully exposed, and they feel that they get the best results from that line of treatment, I have no controversy with them at all. The paper was not written for the sake of controversy, but simply to get at the best method of practice, whatever it may be.—*Dental Review*.

